# Genotype by Environment Interaction on Resistance to Cassava Green Mite Associated Traits and Effects on Yield Performance of Cassava Genotypes in Nigeria

**DOI:** 10.3389/fpls.2020.572200

**Published:** 2020-09-04

**Authors:** Lydia Jiwuba, Agyemang Danquah, Isaac Asante, Essie Blay, Joseph Onyeka, Eric Danquah, Chiedozie Egesi

**Affiliations:** ^1^ Biotechnology Programme, National Root Crops Research Institute, NRCRI, Umudike, Nigeria; ^2^ West Africa Centre for Crop Improvement (WACCI), University of Ghana, Accra, Ghana; ^3^ Department of Crop Science, University of Ghana, Accra, Ghana; ^4^ Department of Plant and Environmental Biology, University of Ghana, Accra, Ghana; ^5^ Department of Plant Breeding and Genetics, Cornell University, Ithaca, NY, United States; ^6^ Cassava Breeding Unit, International Institute for Tropical Agriculture (IITA), Ibadan, Nigeria

**Keywords:** cassava, cassava green mite (CGM), genotype by environment interactions (GEI), additive main effects and multiplicative interaction (AMMI), genotype stability index (GSI), F_1_ hybrid

## Abstract

Cassava is the main source of carbohydrate for over 70% of the people in Nigeria, the world’s largest producer and consumer of the crop. The yields of cassava are, however, relatively low in Nigeria largely due to pests and disease infections that significantly lead to inconsistencies in productivity of cassava genotypes in various environments. Fifty-eight F_1_ hybrid cassava genotypes plus their two parents which served as check varieties were evaluated in three locations for two years (that is six environments). The objectives of the study were to evaluate genotype by environment interactions (GEI) on resistance to cassava green mite [CGM, *Mononychellus tanajoa* (Bondar)] associated traits and effects on yield performance of cassava genotypes in Nigeria and to identify superior genotypes that exhibit high stability which combine CGM resistance and high fresh root yield with general and specific environmental adaptation using additive main effects and multiplicative interaction (AMMI) and genotype stability index (GSI). The combined analysis of variance based on AMMI revealed significant genotype, environment, and genotype by environment interactions (GEI) for all traits. The percentage variation due to environment was higher than the percentage variation due to genotype for cassava green mite severity (CGMS), leaf retention (LR), root dry matter content (RDMC), and fresh root yield (FRY) indicating that environment greatly influenced the expression of these traits. The percentage variation due to GEI accounted for higher percentage variation than that of genotype and environment separately for all traits, indicating the influence of genotype by environment interaction on expression of the traits. These findings reveal that screening/evaluating for these traits needs multi-environment trials. According to GSI ranking, genotypes G31 (IBA131794), G19 (IBA131762), the check variety G52 (TMEB778), and G11 (IBA131748) were identified as the most stable and most resistant to CGM which also combine high FRY and other useful agronomic traits, implying that these traits in cassava can even be incorporated as preferred by farmers. These genotypes can be tested in more environments to determine their adaptability and potential recommendation for release to farmers for growing.

## Introduction

Cassava (*Manihot esculenta*) is a perennial vegetatively propagated woody shrub, mostly grown as an annual that belongs to the Euphorbiaceae family. It is commonly grown in the tropical regions of the world (El-Sharkawy, 2003) with Nigeria being the world’s largest producer with approximately 45 million tonnes (Adekanye et al., 2013). The crop is an essential staple food and animal feed, especially in tropical and sub-tropical Africa because it is a major source of low-cost carbohydrates (Adekanye et al., 2013). Millions of Nigerians (even Africans) daily depend on cassava. It serves as a famine reserve crop, and the plants are left in the soil until required. Cassava is cultivated nearly in all the agro-ecological zones of Nigeria for its edible parts (roots and leaves). This also provides farmers with steady income since it can be harvested at regular intervals.

Unfortunately, biotic and abiotic stresses are the major challenges that farmers face in cassava production. These have resulted in subsequent yield and biomass losses worldwide. Cassava green mite [CGM, *Mononychellus tanajoa* (Bondar)] is one of the major and most destructive dry season cassava pests. CGM threatens food security in Neotropics and Africa by causing yield losses ranging from 30 to 80% (Yaninek and Hanna, 2003). It has been recorded to cause the greatest yield losses in the Americas and Africa (Bellotti et al., 2012), particularly in the seasonally dry areas of the lowland tropics. Reproduction in CGM is arrhenotokous (Roy et al., 2003). There are four active stages, a six-legged larva, two nymphal stages, and the adult stage. From egg to adult developmental stage is about 12.5 days at 27°C. The adult female survives for about 12 days and lays an average of 60 eggs (Yaninek and Hanna, 2003). The adults are green to yellowish in color and can be hardly seen with the naked eyes. The mite pierces and sucks out the fluid content from the abaxial surface of cassava leaves (Yaninek et al., 1989); this causes chlorosis, defoliation, severe ‘candle-stick’ effects with the loss of terminal shoots and die-back.

CGM diminishes the plant’s photosynthetic capacity and growth rate by reducing the leaf area of the plant (Tomkiewicz et al., 1993). Damage by the mite affects the quantity and quality of planting material and roots, reduces the acceptability for both fresh consumption and processing, increases weed infestation, reduces root dry matter, and causes root rot disease in cassava (Yaninek et al., 1989). However, the selection of resistant genotypes to these constraints is difficult due to the complexity of the genotype responses across environments. These differential genotypic responses when exposed to different environments are commonly known as genotype × environment interaction (GEI) (Fox et al., 1997). This kind of interaction leads to bias in the prediction of genetic advance and decreases gain from selection (Farshadfar, 2013). In plant breeding and varietal release programs, GEI enables plant breeders to identify genotypes that are superior with better stability and adaptability (Yan et al., 2000). Various GEI studies on cassava have explored areas related to yield, nutrition, and disease traits (Egesi et al., 2007; Ssemakula and Dixon, 2007; Esuma et al., 2016; Adjebeng-Danquah et al., 2017; Nduwumuremyi et al., 2017; Masinde et al., 2018). Unfortunately, reports are quite few on the effects of GEI on CGM (Bellotti et al., 2012; Chalwe, 2013). In this study, multi-environment trials were conducted to study GEI effects on resistance to CGM and other useful agronomic traits using AMMI model. AMMI analysis is the most reliable statistical method for determining stable cassava clones for specific adaptations, and AMMI biplot analysis enables a simple view of the specific interactions between genotypes and environments (Kvitschal et al., 2009). The AMMI model in multi-environmental trial (MET) data analysis combines analysis of variance (ANOVA) and principal component analysis (PCA) into an integrated approach (Crossa et al., 1990; Gauch and Zobel, 1996). AMMI uses ANOVA to study the main effects of genotypes and environments and a PCA for the residual multiplicative interaction among genotypes and environments. It also helps in grouping environments with the best genotypes into mega-environments using the principal component axis scores and AMMI stability value (ASV) (Hagos and Abay, 2013). The ASV is derived from the Interaction Principal Components Axes 1 and 2 (IPCA1 and IPCA2) scores of the AMMI model (Purchase et al., 2000). Stability parameter alone does not provide much information about the yield performance of a genotype and cannot be used as the only selection parameter since most stable genotypes would not necessarily be the best yield performer. Therefore, Mahmodi et al. (2011) and Tumuhimbise et al. (2014) used yield stability index (YSI) and genotype stability index (GSI) which incorporate high yield performance with stability. Both the YSI and the GSI are based on the sum of the ranking due to ASV scores and yield or performance ranking. Low GSI value indicates desirable genotypes with high mean yield or performance and stability (Mahmodi et al., 2011).

Shoot morphological traits such as high pubescence on the leaves, outstanding retention of the leaves, and ability to stay green also known as cassava green mite associated traits have been reported to promote resistance to CGM; therefore, selecting genotypes for stability and enhanced expression of such traits would improve the durability of resistance and yield (Aina et al., 2007). The main aim of this research was to analyze the effects of genotype by environment interaction on resistance to CGM, shoot morphological and yield traits on 60 cassava genotypes using AMMI model. The specific objectives were to:

1. identify superior genotypes that exhibit high stability which combine CGM resistance and high fresh root yield with general and specific environmental adaptation
2. identify stable genotypes with enhanced expression of the shoot morphological traits to promote resistance to CGM and increase yield
3. identify environments that best represent the target environment for high expression of the traits.

## Materials and Methods

### Study Sites

The research was done at Umudike, Igbariam, and Otobi in two years 2015 and 2016 cropping seasons, totaling six environments (3 locations × 2 years, denoted as Umudike2015, Umudike2016, Igbariam2015, Igbariam2016, Otobi2015, and Otobi2016). Umudike location is situated at 7°24′East, 5°29′North at an altitude of 120 m belonging to humid forest agro ecological zone. It has an annual rainfall of 2,200 mm, average annual temperature of 22 to 31°C and dystric luvisol soils. Igbariam is located in forest-savanna transition agro ecological zone with the geographical coordinates 7°31′East, 5°56′North, altitude of 150 m, annual rainfall of 1,800 mm, average annual temperature of 24 to 32°C and dystric luvisol soils. Otobi is located at 7°20′East and 8°41′North geographical coordinates of southern guinea savanna agro ecological zone. It has an annual rainfall of 1,500 mm, altitude 319 m, mean annual temperature of 24 to 35°C and ferric luvisol soils. These sites represent the country’s major cassava-growing agro-ecological zones.

### Experimental Plant Materials

Sixty cassava genotypes were evaluated in the experiment (**Table 1**). These included fifty-eight F_1_ hybrids and two check varieties (TMEB419 and TMEB778). The fifty-eight F_1_ hybrids were generated by crossing two parents with contrasting responses to CGM; TMEB778 is the female parent, resistant to CGM and high yielding while TMEB419 is the male parent, very susceptible to CGM and moderately yielding. These two parent varieties are extensively used in breeding programs to develop new superior genotypes which combine high and stable yield, pest and disease resistance because of the consumer acceptance qualities of their roots.

**Table 1:** Cassava genotypes with their codes evaluated during 2015 to 2016 cropping seasons at three locations.

Genotype code	Genotype name	Genotype code	Genotype name
G1	IBA131729	G31	IBA131794
G2	IBA131730	G32	IBA131796
G3	IBA131731	G33	IBA131797
G4	IBA131741	G34	IBA131798
G5	IBA131736	G35	IBA131777
G6	IBA131738	G36	IBA131801
G7	IBA131734	G37	IBA131819
G8	IBA131742	G38	IBA131809
G9	IBA131743	G39	IBA131812
G10	IBA131746	G40	IBA131817
G11	IBA131748	G41	IBA131808
G12	IBA131759	G42	IBA131856
G13	IBA131750	G43	IBA131858
G14	IBA131752	G44	IBA131826
G15	IBA131753	G45	IBA131827
G16	IBA131754	G46	IBA131833
G17	IBA131757	G47	IBA131836
G18	IBA131749	G48	IBA131839
G19	IBA131762	G49	IBA131842
G20	IBA131763	G50	IBA131866
G21	IBA131767	G51	IBA131869
G22	IBA131768	G52	TMEB778
G23	IBA131770	G53	IBA131851
G24	IBA131774	G54	IBA131821
G25	IBA131776	G55	IBA131825
G26	IBA131800	G56	IBA131861
G27	IBA131778	G57	IBA131863
G28	IBA131782	G58	TMEB419
G29	IBA131784	G59	IBA131844
G30	IBA131785	G60	IBA131847

G, Genotype.

### Experimental Design and Management

A 6 × 10 alpha lattice design with three replications was used to execute the experiment at each location. The plot area was four ridges with five plants in a ridge giving a population of 20 plants in each plot at a spacing of 1 m × 1 m inter-plant and inter-row distances, respectively. No fertilizer was applied to the trial. Weeds were controlled manually using hoes.

### Data Collection

Data were collected from the inner six plants to avoid border effects. Cassava green mite severity, leaf pubescence, leaf retention, and stay green were evaluated at the peak of dry season (January) at six months after planting (MAP). Harvesting was done at 12 MAP and data were collected on yield and yield traits (**Table 2**).

**Table 2:** Description of the studied traits.

Trait type	Trait name	Abbreviation	Description of trait
Stress trait (categorical)	Cassava green mite severity	CGMS	Cassava Green Mite Severity (CGMS) was evaluated at the visual rating of the damage caused by cassava green mite. Symptoms rated from 1 = highly resistant; no symptoms observed, 2 = resistant; moderate damage, no reduction in leaf size, scattered chlorotic spots on young leaves, 3 = moderately resistant; severe chlorotic symptoms, slight reduction in leaf size, 4 = susceptible; severe chlorotic symptoms and severe reduction in leaf size of young shoot, 5 = highly susceptible; very severe chlorosis, extensive defoliation, candlestick appearance of young shoots.
Morphological (categorical)	Leaf pubescence	LP	Visual rating of the degree of hairiness on the young leaf with 0 = glabrous, 3 = little pubescence, 5 = moderate pubescence and 7 = high pubescence
Morphological (categorical)	Leaf retention	LR	Visual rating of leaf longevity using a scale of 1 = very poor, 2 = poor, 3 = fair, 4 = good, 5 = outstanding
Morphological (categorical)	Stay Green	SG	Visual rating based on leaf longevity and stay green ability using a scale of 1 = poor (<50% of the leaves are live and green), 2 = moderately good (50–74% of the leaves are live and green), 3 = very good (≥75% of the leaves are live and green)
Agronomic (continuous)	Fresh Root Yield	FRY	FRY was estimated according to (Kamau et al., 2011). $\text{FRY (t}/\text{ha)=}\frac{rootweight\left(\frac{kg}{m_{2}}\right)*10000}{1000}$
Agronomic (continuous)	Biomass	Biomass	Total fresh weight of leaves, stem and original planting stake (scale kg)
Agronomic (continuous)	Root Dry Matter Content	RDMC	Determined by the specific gravity method by (Kawano, 1980) (measured in %). $\text{RDMC (\%)=}158.3*\left[\frac{weight\;in\;air}{weight\;in\;air-weight\;in\;water}\right]-142$

### Data Analysis

The effects of the genotype, location, year, genotype by location by year interaction, and replication were determined for each trait in an analysis of variance (ANOVA) using the standard linear model:

$${\text{Y}}_{\text{ijkl}}=\mu +\beta_{i}+{\text{R}}_{\text{ij}}+{\text{G}}_{\text{k}}+\beta_{i}\times \text{Gk}+{\text{e}}_{\text{ijkl}}$$

where *Y_ijkl_* is the phenotypic observations, µ is the mean, β*_i_* is the effect of the location, *R_ij_* is the replication effect, *G_k_* is the clone effect, β*_i_ × G_k_*is the interaction between clone by location, and *e_ijkl_*is the residual. Broad-sense heritability (H^2^) for the traits were captured using the equation $H=\frac{\sigma_{g}^{2}}{\sigma_{g}^{2}+\sigma_{e}^{2}}$ where $\sigma_{g}^{2}$ and $\sigma_{e}^{2}$ are the variance components for the genotype effect and the residual error, respectively, on a plot basis.

The phenotypic correlations were calculated between traits using trait means of the genotypes, and this was performed using Pearson’s correlation coefficient. A multivariate regression was calculated to predict yield traits (FRY, RDMC, and biomass) based on CGM associated traits (CGMS, LP, LR, and SG). The model is

$$Y=a+b_{1}X_{1}+b_{2}X_{2}+b_{3}X_{3}+b_{4}X_{4},$$

where X is the explanatory variable and Y is the dependent variable; b_1_, b_2_ and b_3_ are regression coefficients and a is the intercept (the value of y when x = 0).

Data analyses were done in R statistical software package (R Core Team, 2014) and Genstat 12^th^ edition.

The additive main effect and multiplicative interaction (AMMI) equation by (Mahmodi et al., 2011) was used for the analysis

$$Y_{ij}=\mu +G_{i}+E_{j}+\sum_{k=1}^{n}\lambda_{k}\alpha_{ik}\gamma_{jk}+e_{ij}$$

where *Yij* is the cassava yield of the *i*th genotype in the *j*th environment, *μ* is the grand mean, G*i* and Ej are the *i*th genotypic effect and jth environment effect, respectively, λ_k_ is the square root of the eigenvalue of the PCA axis *k*, α_*ik*_ and γ_*jk*_ are the principal component scores for PCA axis *k* of the *i*th genotype and the *j*th environment, respectively, and *eij* is the residual.

The AMMI model fits the analysis of variance for genotypes and environment effects as the additive main effects and fits the genotype by environment interaction by principal component analysis as the multiplicative terms. AMMI biplots are primarily used in exploring and visualizing G × E pattern.

### Stability Analysis

#### AMMI Stability Value (ASV) Analysis

ASV was calculated for each genotype based on the contributions of the principal component scores (IPCA1 and IPCA2) to the interaction sum of squares (Farshadfar, 2008).

Purchase et al., 2000 proposed ASV formula for calculating and ranking each genotype based on the stability of yield as follows

$$ASV=\sqrt{{\left[\frac{IPCA1_{Sum\;of\;squares}}{IPCA2_{Sum\;of\;squares}}(IPCA1_{score})\right]}^{2}+{\left(IPCA2_{score}\right)}^{2}}$$

Where IPCA1 sum of squares/IPCA2 sum of squares is the weight given to the IPCA1 value by dividing the IPCA1 sum of squares by the IPCA2 sum of squares. Genotypes with lower ASV scores are considered to be more stable across environments (Mahmodi et al., 2011).

#### Genotype Stability Index

Genotype stability index (GSI) was calculated for each genotype based on summing the ranking of overall mean performances for each trait and the ranking for ASV for each trait. GSI incorporates both genotype performance and stability in a single criterion to determine the best-performing stable genotypes across the six environments.

The GSI was calculated as follows:

GSI=RASV+RY;

Where: GSI = genotype stability index for the genotype across environments for each trait; RASV = rank of ASV across environments; RY = rank of the genotypes based on mean performance of a trait across environments. The genotype with the lowest GSI score for a particular trait was considered as the best for combined performance and stability across the environments (Farshadfar et al., 2012).

## Results

### Descriptive Summary of Cassava Traits Analyzed Across the Environments

Summary statistics of phenotypic data acquired for two cropping seasons 2015 and 2016 in the three locations Igbariam, Otobi, and Umudike are shown in **Table 3**.

**Table 3:** Summary statistics (mean ± standard error of mean (SEM), coefficient of variation (CV) and broad-sense heritability estimates (H^2^) for phenotypic data across locations and years.

Trait	Igbariam			Otobi			Umudike			Pooled		
	Mean ± SEM	CV	H^2^	Mean ± SEM	CV	H^2^	Mean ± SEM	CV	H^2^	Mean ± SEM	CV	H^2^
CGMS	2.04 ± 0.05	49.49	0.37	2.80 ± 0.07	51.19	0.32	1.72 ± 0.05	52.39	0.39	2.19 ± 0.04	55.94	0.36
LP	4.65 ± 0.14	58.29	0.27	3.36 ± 0.15	87.13	0.36	4.74 ± 0.14	55.17	0.47	4.25 ± 0.09	66.42	0.37
LR	4.00 ± 0.05	24.28	0.45	3.18 ± 0.07	44.59	0.52	4.05 ± 0.05	25.25	0.53	3.75 ± 0.03	32.60	0.50
SG	2.46 ± 0.03	22.44	0.33	2.14 ± 0.04	34.48	0.28	2.55 ± 0.03	23.30	0.36	2.39 ± 0.02	27.55	0.32
BIOMASS	8.76 ± 0.36	77.65	0.66	6.34 ± 0.31	91.63	0.58	10.97 ± 0.46	78.94	0.76	8.69 ± 0.23	85.46	0.67
RDMC	28.46 ± 0.30	20.29	0.39	24.00 ± 0.40	31.92	0.24	30.85 ± 0.33	20.49	0.41	27.77 ± 0.22	25.96	0.35
FRY	21.12 ± 0.71	48.10	0.47	18.01 ± 0.60	62.78	0.59	24.98 ± 0.92	49.77	0.62	21.37 ± 0.48	58.85	0.56

CGMS, Cassava green mite severity; LP, leaf pubescence; LR, leaf retention; SG, stay green; RDMC, root dry matter content; FRY, fresh root yield; SEM, standard error of mean; CV, coefficient of variation; H^2^, broad-sense heritability.

The lowest mean (1.72 of a maximum of 5) was obtained for CGM at Umudike, and the highest mean (2.80 of a maximum of 5) was obtained for CGM at Otobi. The highest mean for LP, LR, and SG was found in Umudike, whereas the lowest was found in Otobi. This means that the higher the CGM attack on the leaves, the lower the LP, LR, and SG. The highest mean for biomass (10.97), FRY (24.98), and RDMC (30.85) was recorded at Umudike and the lowest mean for biomass (6.34), FRY (18.01), and RDMC (24.0) was recorded at Otobi. Broad-sense heritability (H^2^) estimates varied from low-to-moderate, with the highest H^2^ for biomass (0.67) and the lowest H^2^ for SG (0.32). Coefficient of variation was used to measure the variability among the traits, ranging from 25.96% for RDMC to 85.46% for biomass.

### Correlation Analysis of the Traits

The results of phenotypic correlation between CGMS and other useful agronomic traits (**Table 4**) showed that LP (r = −0.80), LR (r = −0.52), SG (r = −0.52), FRY (r = −0.50), RDMC (r = −0.20), and biomass (r = −0.31) were significantly and negatively correlated with CGMS (P < 0.001, P < 0.01, and P < 0.05). LR (r = 0.57), SG (r = 0.61), and FRY (r = 0.44) significantly and positively correlated with LP. LR had a significant positive correlation with SG (r = 0.76) and FRY (r = 0.44). FRY had a significant positive correlation with SG (r = 0.31), RDMC (r = 0.32), and biomass (r = 0.28). These results indicate that plants with severe CGM had glabrous to little leaf pubescent, poor leaf retention and stay green, low root dry matter content, low root and stem yield.

**Table 4:** Phenotypic correlation coefficients for cassava green mite severity and other agronomic traits.

	CGMS	LP	LR	SG	FRY	RDMC	BIOMASS
CGMS	1	−0.80^***^	−0.52^***^	−0.52^***^	−0.50^***^	−0.20^*^	−0.31^**^
LP		1	0.57^***^	0.61^***^	0.44^***^	0.12^ns^	0.14^ns^
LR			1	0.76^***^	0.32^**^	0.19^ns^	0.18^ns^
SG				1	0.31^**^	0.14^ns^	0.14^ns^
FRY					1	0.32^**^	0.34^**^
RDMC						1	0.28^*^
BIOMASS							1

CGMS, Cassava green mite severity; LP, leaf pubescence; LR, leaf retention; SG, stay green; RDMC, root dry matter content;, FRY, fresh root yield.

***significant at P < 0.001, **significant at P < 0.01, *significant at P < 0.05, ns, not significant.

### Multivariate Regression Analysis

A multivariate regression was calculated to predict yield traits (FRY, RDMC, and biomass) based on CGM associated traits (CGMS, LP, LR, and SG). For FRY, a high significant regression equation was found [F (4, 1075) = 73.48, p < 0.000], with an R^2^ of 0.21. Using the same variables, multivariate regression model was carried out for RDMC and biomass. The multivariate regression model for RDMC was highly significant, R^2^ = 0.06, F (4, 1075) = 27.63, p < 0.000. For biomass, the model was highly significant at the p < 0.000 level, R^2^ = 0.10, F (4, 1075) = 29.38. This model showed significant negative sign of CGMS on the yield traits. This means that the CGMS had significant negative effect on FRY, RDMC, and biomass. The results of the model showed that CGMS caused a loss of 4.5 tonnes of fresh root yield per ha (**Supplementary Table 1**). The current value of this loss is around 367.85 USD (135,000 Naira). In addition, the results showed that LR is significantly positive for FRY. Also, for biomass, LP (B = −0.27, p < 0.01) and LR (B = 0.61, p < 0.01) contributed significantly to the model. For DMC, LP (B = −0.25, p < 0.01) and LR (B = 0.98, p < 0.000) contributed significantly to the model.

### Analysis of Variance for Traits Evaluated

The combined analysis of variance results for all traits evaluated in three locations across two years revealed highly significant (P < 0.001) for effects of genotype, location, and genotype by location interaction, indicating that there were substantial variations in phenotypic response across locations, and also the locations had a strong influence on the traits (**Supplementary Table 2**). Genotype by year interaction and location by year interaction were not significant for any of the traits. Besides SG, all other traits were significant for year effect, genotype by location by year interaction and location by rep. The results also indicated that the effect of replication was significant (P < 0.001 and 0.05, respectively) for CGMS, LP, LR, biomass, RDMC, and FRY. In other words, blocking was effective for these traits.

### AMMI Analysis of Variance

The results of the combined AMMI analysis of variance revealed high significant (P < 0.001 and P < 0.01) effects of genotypes, environment, and genotype by environment interactions for all the traits evaluated (see **Table 5**). Block effect was highly significant (P < 0.001) for CGMS, LP, biomass, RDMC, and FRY and very significant (P < 0.01) for LR but was not significant for SG. The % treatment sum of squares due to environment was higher than the % treatment sum of squares due to genotype for CGMS, LR, RDMC, and FRY, indicating that environment greatly influenced the expression of these traits. The interaction principal component analysis (IPCA1) indicated high significant (P < 0.0001) variation for all traits. IPCA2 also showed significant difference (P < 0.001 and P < 0.05) for all the traits, justifying the use of the AMMI2 (IPCA2 *vs* IPCA1) biplot model for all the traits studied. AMMI2 biplots show the pattern of the genotype by environment interaction based on the plot of the IPCA1 and IPCA2 of both genotype and environment.

**Table 5:** Combined AMMI ANOVA for 7 traits of 60 cassava genotypes evaluated across six environments.

Source	Mean squares							
	df	CGMS	LP	LR	SG	BIOMASS	RDMC	FRY
Treatments	359	2.49^***^	10.03^***^	2.43^***^	0.61^**^	94.10^***^	78.80^**^	448.00^***^
Genotypes (G)	59	3.69^***^	15.34^***^	2.65^*^	0.80^***^	184.70^***^	80.70^***^	628.00^***^
Environments (E)	5	45.35^***^	93.44^**^	35.99^***^	6.90^***^	807.20^***^	1,929.20^***^	10,679.00^***^
Block	12	5.93^***^	27.03^***^	2.73^**^	0.43^ns^	180.90^***^	104.00^***^	672.00^***^
Interactions (GxE)	295	1.52^***^	7.55^*^	1.82^***^	0.47^***^	63.90^***^	47.10^**^	239.00^***^
IPCA1	63	3.99^***^	16.33^***^	5.35^***^	1.20^***^	138.50^***^	88.40^***^	418.00^***^
IPCA2	61	1.42^***^	7.21^**^	1.30^***^	0.36^**^	69.90^***^	56.00^***^	320.00^***^
Error	708	0.92	6.59	1.00	0.34	33.30	37.50	145.00
	**Sum of squares**							
**Source**	**df**	**CGM**	**LP**	**LR**	**SG**	**BIOMASS**	**RDMC**	**FRY**
Treatments	359	892.90	3,600.00	872.00	219.80	33,793.00	28,290.00	160,982.00
Genotypes (G)	59	217.80	905.00	156.50	47.10	10,895.00	4,760.00	37,080.00
Environments (E)	5	226.70	467.00	180.00	34.50	4036.00	9,646.00	53,394.00
Block	12	71.10	324.00	32.70	5.20	2170.00	1,248.00	8,058.00
Interactions (GxE)	295	448.40	2,227.00	535.50	138.20	18,862.00	13,884.00	7,0507.00
IPCA1	63	251.30	1,029.00	337.30	75.50	8,726.00	5,571.00	26,339.00
IPCA2	61	86.80	440.00	79.50	21.70	4,262.00	3,417.00	19,520.00
Error	708	649.60	4,663.00	705.30	240.80	23,547.00	26,553.00	102,609.00
% treatment SS due to G		24.39	25.14	17.95	21.43	32.24	16.83	23.03
% treatment SS due to E		25.39	12.97	20.64	15.70	11.94	34.10	33.17
% treatment SS due to GxE		50.22	61.86	61.41	62.88	55.82	49.08	43.80
% GxE SS due to IPCA1		56.04	46.21	62.99	54.63	46.26	40.13	37.36
% GxE due to IPCA2		19.36	19.76	14.85	15.70	22.60	24.61	27.69

IPCA1, Interaction principal component axis 1; IPCA2, Interaction principal component axis 2; CGMS, Cassava green mite severity; LP, leaf pubescence; LR, leaf retention; SG, stay green; RDMC, root dry matter content; FRY, fresh root yield. ***significant at P < 0.001, **significant at P < 0.01, *significant at P < 0.05, ns, not significant.

The % treatment sum of squares due to GEI for CGMS was higher (50.22%) than that due to environment (25.39%) and genotype (24.39%). The GEI variation partitioned into principal components indicated that IPCA1 accounted for 56.04% and IPCA2, 19.36% (**Table 5**). For leaf pubescence, 25.14% of the treatment sum of squares was due to genotype effect, while environment and interaction effect accounted for 12.97 and 61.86%, respectively. The IPCA1 accounted for 46.21% with IPCA2 accounting for 19.76%. A greater proportion of the treatment sum of squares for leaf retention was due to interaction effect (61.41%) followed by environment effect (20.64%) and genotype effect (17.95%). The first two interaction principal component axes (IPCA1 and IPCA2) cumulatively accounted for 77.83% of the GE interaction SS. Genotype effects accounted for 21.43% of the treatment sum of squares for stay green ability, whereas environment and interaction effects accounted for 15.70 and 62.88%, respectively. The IPCA1 accounted for 54.63% of the interaction sum of squares with IPCA2 accounting for 15.70%. For biomass, interaction effect (55.82%) contributed a greater proportion of the treatment sum of squares compared with genotype effect (32.24%) and environment effect (11.94%). The IPCA1 and IPCA2 explained 46.26 and 22.60% of GE interaction SS, respectively. The influence of genotype by interaction (49.08%) on dry matter content of the root was greater than both effects of environment (34.10%) and genotype (16.83%). The IPCA1 and IPCA2 accounted for 64.74% of the interaction sum of squares. Interaction effect (43.80%) on fresh root yield was greater than the effect of environment (33.17%) and genotype (23.03%). The first two principal component axes captured a total of 65.04% of the interaction SS. The % treatment sum of squares due to GEI accounted for higher % variation than that for genotype and environment separately for all traits studied, indicating that all the observed variations cannot be explained independently by each variable.

### Stability and Biplot Analysis

#### Genotype-by-Environment Interaction Effects on Resistance to Cassava Green Mite Across Environments

**Figure 1:**
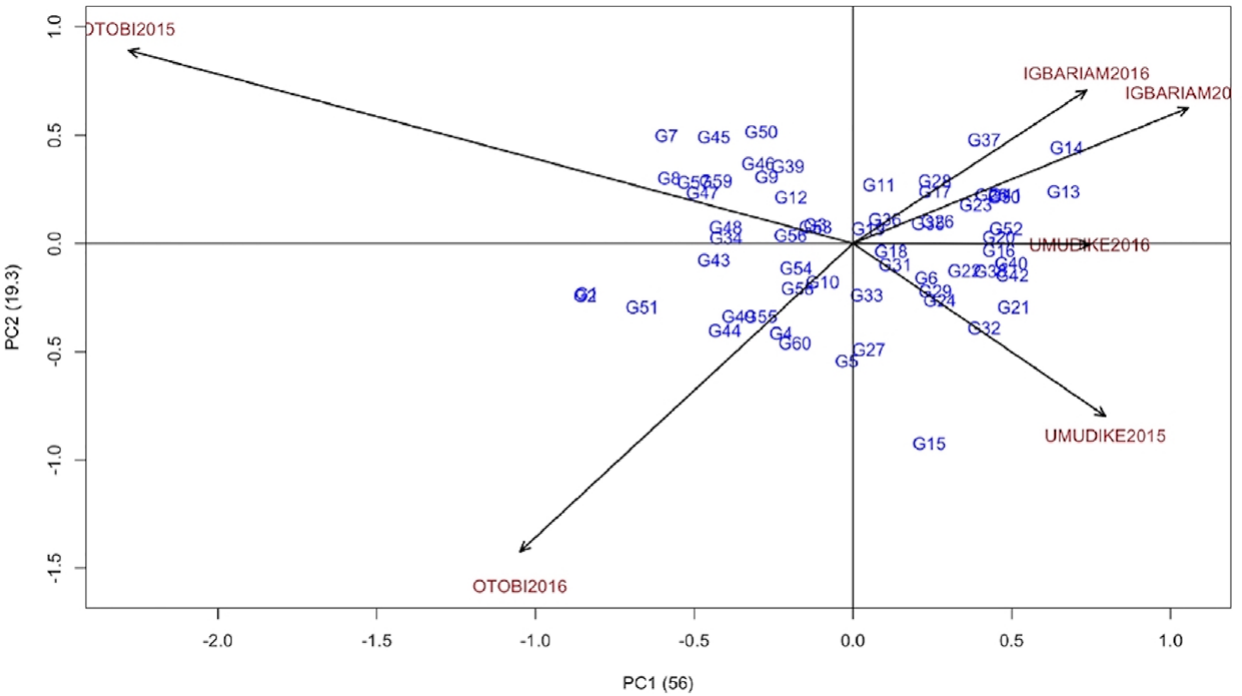
AMMI2 biplot for CGMS.

The majority of the genotypes were scattered far from the AMMI2 biplot origin, indicating that most of the genotypes were unstable across the six environments (**Figure 1**). The ASV and the AMMI2 biplots indicated that G19, G33, G36, G10, and G18 were the most stable genotypes but not those showing the lowest severity and should therefore not be recommended, whereas the GSI revealed that G31, G19, G52, G38, and G11 showed the lowest severity and were the most stable genotypes (**Table 6**). Thus, genotypes G31 and G19 were more stable with better response to CGM attack than the check variety (G52). G19, G18, G31, and G36 lie close to the biplot origin (0, 0) indicating low interaction with the environments scattered far from the biplot center (**Figure 1**). The mean rank and GSI rank show that G31 was the best genotype to resist cassava green mite attack (**Table 6**). The AMMI2 biplot indicated that the environments Otobi2015 and Otobi2016 were located far away from the biplot origin thus elicit a stronger interactive force than those environments Umudike2016, Umudike2015, Igbariam2015 and Igbariam2016 which were near the origin. The distance from the biplot origin (0, 0) indicates that G37, G14, and G13 were positively interacting with Igbariam2016 and Igbariam2015, indicating specific adaptation to these environments, whereas genotype G15 showed specific adaptation to the Umudike2015 environment. Environment Umudike2016 which had the lowest CGMS score appears to be the most stable environment with genotypes G52 (check variety), G20, and G16. G1, G2, and G51 showed specific adaptation to the Otobi2015 environment, and G7 and G8 showed positive interaction with the Otobi2015 environment, indicating specific adaptation (**Figure 1**).

**Table 6:** Overall mean and ranking of 60 cassava genotypes across six environments based on cassava green mite score.

Genotype	ASV	GSI	ASV rank	GSI rank	CGMS means
G1	2.44	104.5	59	46	2.61
G10	0.33	42.5	4	39	2.44
G11	0.36	13	8	5	1.61
G12	0.6	42	15	27	2.17
G13	1.94	63	56	7	1.67
G14	2	75	58	17	1.78
G15	1.16	64.5	33	32	2.28
G16	1.33	51	44	7	1.61
G17	0.79	57	23	34	2.28
G18	0.35	24	5	19	1.83
G19	0.16	3	1	2	1.56
G2	2.46	103.5	60	44	2.56
G20	1.33	52	43	9	1.72
G21	1.5	80	53	27	1.94
G22	1.4	62.5	48	15	1.72
G23	1.13	52.5	32	21	1.89
G24	0.83	36	25	11	1.72
G25	1.28	69	40	29	2.17
G26	0.78	39	21	18	1.78
G27	0.51	48.5	10	39	2.33
G28	0.8	38.5	24	15	1.78
G29	0.78	42.5	22	21	1.89
G3	0.36	30	7	23	1.89
G30	1.4	78.5	47	32	2.22
G31	0.4	10	9	1	1.5
G32	1.26	48	37	11	1.72
G33	0.27	13	2	11	1.72
G34	1.16	89	34	55	2.83
G35	0.69	40	17	23	1.89
G36	0.31	26	3	23	1.89
G37	1.29	69	42	27	2.06
G38	1.26	42	38	4	1.56
G39	0.69	49.5	18	32	2.17
G4	0.77	58.5	20	39	2.33
G40	1.45	64.5	50	15	1.78
G41	1.41	56	49	7	1.61
G42	1.46	89.5	51	39	2.33
G43	1.27	92	39	53	2.78
G44	1.24	91	36	55	2.78
G45	1.36	76.5	45	32	2.17
G46	0.94	53	28	25	1.89
G47	1.39	89.5	46	44	2.56
G48	1.16	70	35	35	2.28
G49	1.1	69.5	31	39	2.33
G5	0.55	54	12	42	2.5
G50	0.98	87	29	58	2.89
G51	1.94	102.5	57	46	2.61
G52	1.02	33	30	3	1.56
G53	0.35	55.5	6	50	2.67
G54	0.53	71	11	60	3.17
G55	0.91	76.5	27	50	2.61
G56	0.57	63.5	14	50	2.67
G57	1.48	110	52	58	2.94
G58	0.55	71	13	58	3
G59	1.29	79.5	41	39	2.44
G6	0.69	30.5	16	15	1.72
G60	0.7	74	19	55	2.78
G7	1.77	104.5	55	50	2.67
G8	1.71	103.5	54	50	2.72
G9	0.85	75.5	26	50	2.61

ASV, AMMI stability value; G, Genotype; GSI, genotype selection index; CGMS, Cassava green mite severity.

#### Genotype-by-Environment Interaction Effects on Shoot Morphological Traits Across the Six Environments

**Figure 2:**
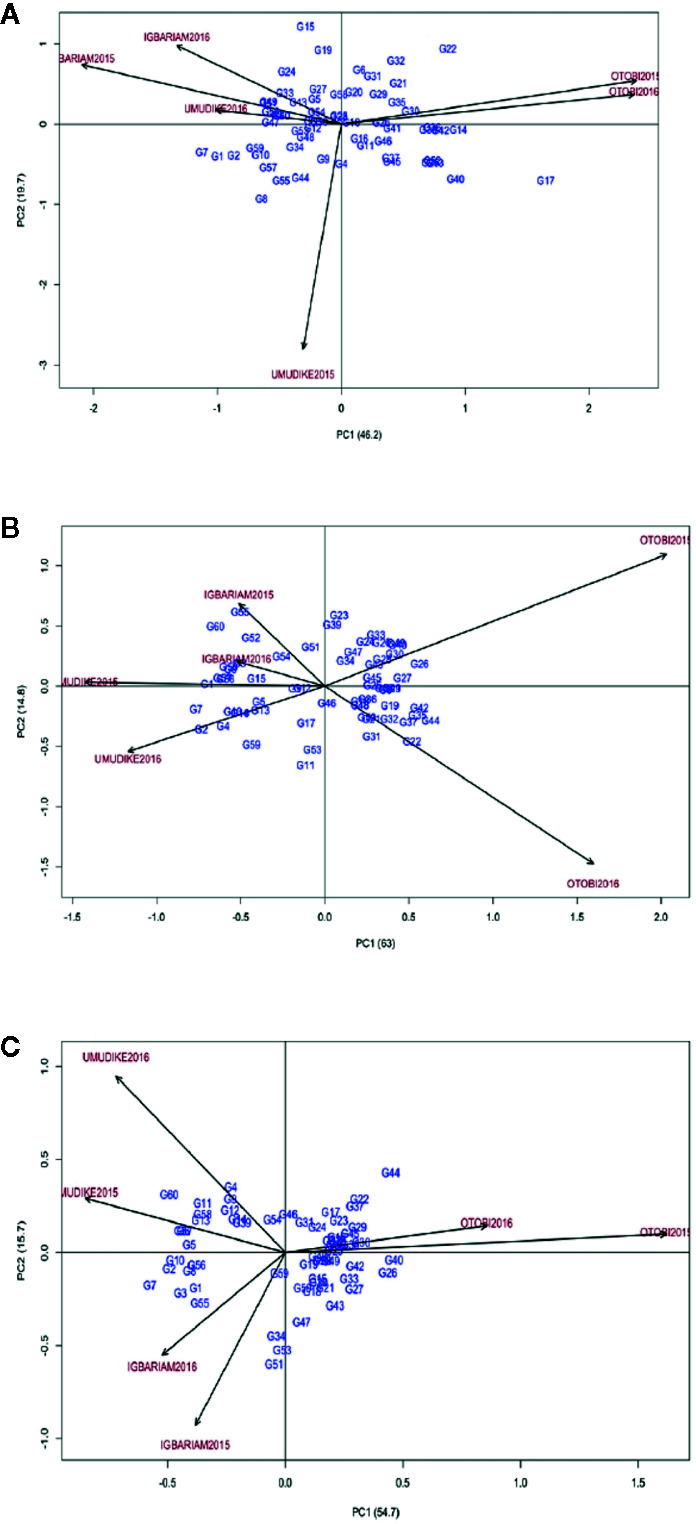
AMMI2 biplot. **(A)** for LP, **(B)** for LR, **(C)** for SG.

For LP, these genotypes G28, G23, G18, G58, and G19 were the best performers in that order since they have the least scores for ASV (**Table 7**). G17, G7, G1, G40, and G22 were ranked in that order as the least stable genotypes. GSI ranked G31, G19, G52 (check variety), G11, and G41 as genotypes with highest pubescent and improved stability (**Table 7**). The AMMI2 biplot graph showed that apart from environment Umudike2016, all other environments were far from the biplot origin (0, 0). It also revealed that G28, G23, and G18 were close to the origin, and hence they were most stable genotypes (**Figure 2A**), whereas G15, G22, and G17 were found to be more responsive because they are far from the origin. Genotypes that are close to each other appear to have similar performance, and those close to the environment have better adaptation to that specific environment. In this case, the best adapted genotypes for environments Otobi2016 and Otobi2015 were G22, G17, and G32; for environment Igbariam2015 were G7, G24, and G33, for Igbariam2016 were G24, G19, and G33; for environment Umudike2016 was G7; for environment Umudike2015 were G8 and G7. The AMMI2 biplots for LR and SG showed that most of the genotypes were scattered far from the biplot center, implying that most genotypes were unstable (**Figures 2B, C**). In **Figure 2B** for LR, G46, G12, G34, and G51 lie close to the biplot origin (0, 0) revealing weak interaction with the locations scattered far from the biplot center. The mean and GSI ranks indicate that G31, G45, G44, G46, and G19 were the best five genotypes with better response to leaf retention and improved (**Table 7**). On the genotype points on environments, G26 had positive interaction with the Otobi2015 environment, indicating specific adaptation to the environment. G60 and G55 showed specific adaptation to the Igbariam2015, while G7 and G2 were specifically adapted to the Umudike2016 and Umudike2015 environments, revealing their specific adaptation to both environments. G22, G31, G37, and G44 were specifically adapted to Otobi2016, whereas G3 had a positive interaction with Igbariam2016 environment. For SG, G59, G46, and G54 were close to the biplot origin (0, 0) indicating general adaptation to the environments while G7, G60, and G44, which were far apart from the origin, tend to show a dissimilar pattern of response over the environments (**Figure 2C**). Therefore, the genotypes close to the origin are not sensitive to environmental interaction, while those that are distant from the origin are sensitive and have a wider interaction. To identify superior genotypes with very good stay green ability and high stability, GSI was used to rank these genotypes G31, G19, G45, G46, and G48 as the superior genotypes across environments.

**Table 7:** Overall mean and ranking of 60 cassava genotypes across six environments for shoot morphological traits.

Genotype	LP					Genotype	LR					Genotype	SG				
	ASV	GSI	ASV rank	GSI rank	LP mean		ASV	GSI	ASV rank	GSI rank	LR mean		ASV	GSI	ASV rank	GSI rank	SG mean
G1	2.37	96.5	58	38.5	3.94	G1	2.96	115	58	57	3.17	G1	1.34	98	48	50	2.17
G10	1.57	91.5	45	46.5	3.61	G10	2.33	81.5	47	34.5	3.61	G10	1.63	111	57	54	2.11
G11	1.09	34	30	4	5.56	G11	0.81	38	11	27	3.83	G11	1.25	84	44	40	2.28
G12	0.54	55	11	44	3.78	G12	0.57	40.5	6	34.5	3.61	G12	0.85	64	31	33	2.33
G13	1.83	79.5	52	27.5	4.61	G13	1.64	81.5	28	53.5	3.28	G13	1.27	85	45	40	2.28
G14	2.21	86	55	31	4.28	G14	2.15	91.5	42	49.5	3.33	G14	0.73	84	24	60	1.89
G15	1.39	89	41	48	3.56	G15	1.72	93	34	59	3.11	G15	0.5	66	12	54	2.11
G16	0.98	42	26	16	4.89	G16	2.15	92.5	43	49.5	3.33	G16	0.77	32	26	6	2.67
G17	3.93	105	60	45	3.67	G17	0.56	53.5	4	49.5	3.33	G17	0.71	62	22	40	2.28
G18	0.19	19	3	16	4.89	G18	0.9	22.5	12	10.5	4.17	G18	0.45	18.5	7	11.5	2.61
G19	0.39	7.5	5	2.5	5.67	G19	1.26	27	22	5	4.28	G19	0.68	22	20	2	2.72
G2	2.07	87	54	33	4.22	G2	3.13	100	59	41	3.5	G2	1.72	116	58	57.5	2.06
G20	0.47	20.5	7	13.5	4.94	G20	1.48	54	25	29	3.83	G20	0.52	35	13	22	2.5
G21	1.2	71.5	33	38.5	3.94	G21	1.21	50.5	20	30.5	3.72	G21	0.62	51	18	33	2.33
G22	2.22	63.5	56	7.5	5.22	G22	2.24	56.5	46	10.5	4.17	G22	1.14	53.5	42	11.5	2.61
G23	0.12	32	2	30	4.33	G23	0.69	53	8	45	3.44	G23	0.81	63	30	33	2.33
G24	1.22	58.5	34	24.5	4.72	G24	1.09	39	15	24	3.89	G24	0.49	38.5	11	27.5	2.44
G25	1.75	85	49	36	4.11	G25	1.2	51	19	32	3.67	G25	0.72	63	23	40	2.28
G26	0.75	36	17	19	4.83	G26	2.4	66.5	51	15.5	4.11	G26	1.52	69	53	16	2.56
G27	0.62	55.5	15	40.5	3.89	G27	1.97	85	40	45	3.44	G27	1.04	84	38	46	2.22
G28	0.1	12	1	11	5.06	G28	1.47	46	24	22	3.94	G28	0.54	37	15	22	2.5
G29	0.8	29	20	9	5.11	G29	1.69	46.5	33	13.5	4.17	G29	1.08	62	40	22	2.5
G3	1.23	59.5	35	24.5	4.72	G3	2.16	81.5	44	37.5	3.56	G3	1.57	90	54	36	2.33
G30	1.32	90.5	37	53.5	3.22	G30	1.78	76	35	41	3.5	G30	1.13	87	41	46	2.22
G31	1.3	37	36	1	5.83	G31	1.69	33	32	1	4.5	G31	0.33	7	5	2	2.72
G32	0.84	28	22	6	5.28	G32	1.65	37.5	30	7.5	4.22	G32	0.7	27	21	6	2.67
G33	1.14	43	31	12	5	G33	1.36	43.5	23	20.5	3.94	G33	0.96	61.5	34	27.5	2.44
G34	0.91	70.5	24	46.5	3.61	G34	0.57	58.5	5	53.5	3.28	G34	0.47	31	9	22	2.5
G35	0.53	17.5	10	7.5	5.22	G35	2.35	58.5	48	10.5	4.17	G35	0.77	43	27	16	2.56
G36	1.72	69	48	21	4.83	G36	1.09	31.5	14	17.5	4.06	G36	0.75	47	25	22	2.5
G37	1.03	47	28	19	4.83	G37	2.13	46	41	5	4.28	G37	1.07	50.5	39	11.5	2.61
G38	1.64	65	46	19	4.83	G38	1.5	33.5	26	7.5	4.22	G38	0.96	46.5	35	11.5	2.61
G39	0.55	34.5	12	22.5	4.78	G39	0.55	30	3	27	3.83	G39	0.66	35	19	16	2.56
G4	0.49	42	9	33	4.22	G4	2.58	110	54	56	3.22	G4	0.88	90.5	33	57.5	2.06
G40	2.26	90	57	33	4.22	G40	1.87	52.5	37	15.5	4.11	G40	1.61	84.5	55	29.5	2.39
G41	0.96	30	25	5	5.33	G41	1.65	46.5	29	17.5	4.06	G41	0.78	39.5	28	11.5	2.61
G42	1.87	79	53	26	4.61	G42	2.4	74	50	24	3.89	G42	1.04	77	37	40	2.28
G43	0.86	50.5	23	27.5	4.61	G43	1.25	31.5	21	10.5	4.17	G43	0.79	35	29	6	2.67
G44	1.02	82	27	55	3.06	G44	2.68	59	56	3	4.33	G44	1.62	67.5	56	11.5	2.61
G45	1.06	42.5	29	13.5	4.94	G45	1.2	20	18	2	4.44	G45	0.97	38	36	2	2.72
G46	0.82	31	21	10	5.11	G46	0.15	6	1	5	4.28	G46	0.21	8	2	6	2.67
G47	1.33	88	38	50	3.39	G47	0.78	40.5	10	30.5	3.72	G47	0.45	41	8	33	2.33
G48	0.69	58.5	16	42.5	3.83	G48	0.9	26.5	13	13.5	4.17	G48	0.49	16	10	6	2.67
G49	1.4	71	42	29	4.39	G49	1.83	56.5	36	20.5	3.94	G49	0.35	28	6	22	2.5
G5	0.6	70	14	56	2.94	G5	1.67	90	31	59	3.11	G5	1.42	103	49	54	2.11
G50	0.42	48.5	6	42.5	3.83	G50	1.1	40	16	24	3.89	G50	0.32	26	4	22	2.5
G51	1.41	83.5	43	40.5	3.89	G51	0.48	36.5	2	34.5	3.61	G51	0.62	46.5	17	29.5	2.39
G52	1.77	53.5	51	2.5	5.67	G52	1.91	85	38	47	3.44	G52	0.54	38	16	22	2.5
G53	0.78	56	19	37	4.11	G53	0.61	48	7	41	3.5	G53	0.52	47	14	33	2.33
G54	0.49	68	8	60	1.5	G54	1.12	76	17	59	3.11	G54	0.26	53	3	50	2.17
G55	1.33	90.5	39	51.5	3.33	G55	2.22	98.5	45	53.5	3.28	G55	1.29	92	46	46	2.22
G56	1.34	97	40	57	2.61	G56	2.5	94	53	41	3.5	G56	1.31	93	47	46	2.22
G57	1.48	97.5	44	53.5	3.22	G57	2.58	104.5	55	49.5	3.33	G57	1.52	91.5	51.5	40	2.28
G58	0.38	63	4	59	1.78	G58	2.43	105.5	52	53.5	3.28	G58	1.24	93	43	50	2.17
G59	1.65	96	47	49	3.56	G59	1.91	66	39	27	3.83	G59	0.14	47	1	46	2.22
G6	0.76	34	18	16	4.89	G6	1.53	46	27	19	4	G6	1.52	91.5	51.5	40	2.28
G60	1.14	90	32	58	2.5	G60	2.8	98	57	41	3.5	G60	1.75	118	59	59	2
G7	2.66	81.5	59	22.5	4.78	G7	3.25	94.5	60	34.5	3.61	G7	2.01	82	60	22	2.5
G8	1.77	101.5	50	51.5	3.33	G8	2.39	94	49	45	3.44	G8	1.42	104	50	54	2.11
G9	0.55	48	13	35	4.17	G9	0.75	46.5	9	37.5	3.56	G9	0.86	86	32	54	2.11

ASV, AMMI stability value; G, Genotype; GSI, genotype selection index; LP, leaf pubescence; LR, leaf retention; SG, stay green.

#### Genotype-by-Environment Interaction Effects on Yield Traits Across the Six Environments

**Figure 3:**
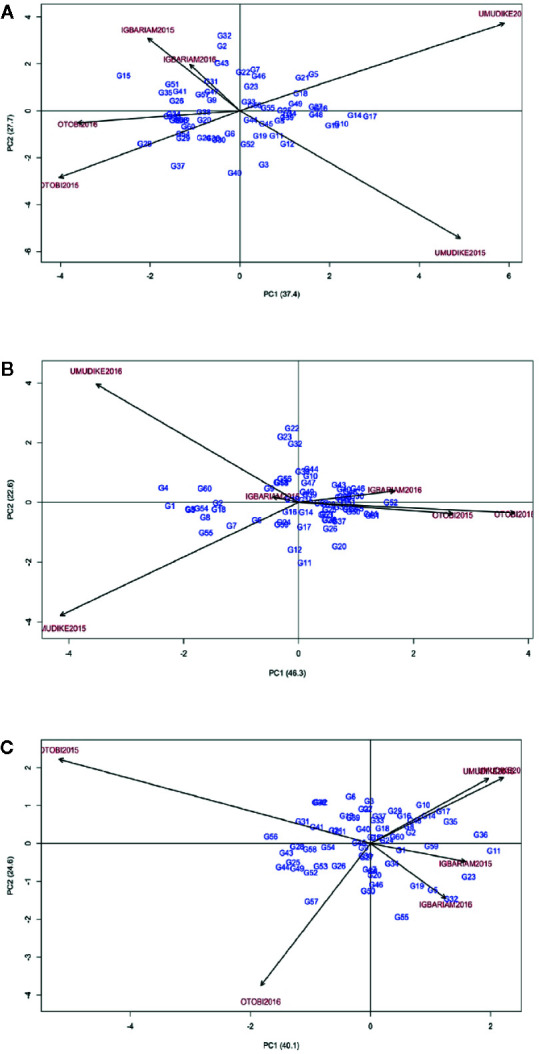
AMMI2 biplot **(A)** for FRY, **(B)** for biomass, **(C)** for RDMC.

For FRY, the AMMI2 biplot and ASV indicated that the most stable genotypes were G33, G44, G56, and G55, whereas the most unstable ones were G15, G32, and G28 (**Figure 3A**, **Table 8**). Particularly, ASV measures genotype by environment interaction effects but does not show the best (high-yielding and stable) genotype. The GSI combines both genotype stability and high yield, indicating the best method to determine ideal genotypes. Accordingly, GSI ranked genotypes G52, G31, G11, G19, and G25 as the most ideal genotypes for all environments (**Table 8**). The check variety (G52) was, therefore, the highest yielder in all the environments. In **Figures 3A–C**, the environmental scores are joined to the origin by side lines. Environments with long spokes exert stronger interactive forces than those with short spokes. From the point where the environments are connected to the origin, the environments Igbariam2015 and Igbariam2016 had short spokes, and they exert weak interactive forces (**Figure 3A**). The AMMI biplot and ASV indicated that G19, G15, G14, G16, and G49 were the most stable genotypes (**Figure 3B**, **Table 8**), whereas the GSI showed that G9, G1, G3, G60, and G54 were the most ideal genotypes for biomass. For RDMC, ASV ranking and AMMI biplots indicated genotypes G15, G9, G12, G45, and G30 were most stable, whereas the GSI indicated that genotypes G15, G19, G52 (check variety), G9, and G37 were the ideal (**Figure 3C**, **Table 8**). Based on the distance from the biplot origin (0, 0), the highest GE interaction for RDMC was recorded in the Otobi2015 and Otobi2016 environments (**Figure 3C**).

**Table 8:** Overall mean and ranking of 60 cassava genotypes across six environments for yield and yield traits.

Genotype	Biomass					Genotype	RDMC					Genotype	FRY				
	ASV	GSI	ASV rank	GSI rank	biomass mean		ASV	GSI	ASV rank	GSI rank	RDMC mean		ASV	GSI	ASV rank	GSI rank	FRY mean
G1	4.58	61	59	2	15.51	G1	0.84	28	18	10	29.81	G1	1.45	22	20	2	37.5
G10	0.98	28	15	13	11.54	G10	1.75	57	42	15	29.23	G10	3.1	66	55	11	32.85
G11	2.03	65	40	25	9.17	G11	3.36	96	60	36	27.4	G11	1.53	25	22	3	34.95
G12	1.59	71	30	41	6.41	G12	0.23	51	3	48	26.05	G12	2.01	49	39	10	32.93
G13	0.93	29	12	17	10.71	G13	0.98	38	22	16	29.23	G13	2.85	62	53	9	33.08
G14	0.42	38	3	35	7.29	G14	1.74	46	40	6	30.86	G14	3.46	71	58	13	32.39
G15	0.27	61	2	59	4.11	G15	0.2	2	1	1	32.93	G15	3.81	110	59	51	20.41
G16	0.45	41	4	37	7.22	G16	1.8	73	44	29	27.97	G16	2.43	55	49	6	34.04
G17	0.84	38	9	29	8.41	G17	2.15	62	50	12	29.59	G17	3.92	80	60	20	30.69
G18	2.86	60	50	10	12.82	G18	1.68	50	39	11	29.61	G18	1.97	56	34	22	29.81
G19	0.26	13	1	12	12.28	G19	0.51	11	9	2	31.59	G19	1.23	31	12	19	30.76
G2	2.88	62	51	11	12.28	G2	1.13	36	28	8	30.1	G2	2.82	60	52	8	33.32
G20	2.06	69	41	28	8.64	G20	0.84	62	17	45	26.57	G20	1.14	55	10	45	22.75
G21	1.09	64	19	45	6.06	G21	1.01	66	23	43	26.75	G21	2.36	79	46	33	26.48
G22	2.5	69	46	23	9.4	G22	0.92	58	21	37	27.36	G22	1.65	42	26	16	31.45
G23	2.26	65	45	20	10.33	G23	2.81	116	58	58	23.91	G23	1.09	45	9	36	26.24
G24	0.85	58	10	48	5.89	G24	0.45	67	8	59	23.51	G24	1.58	52	24	28	27.62
G25	1.12	74	20	54	5.19	G25	2.16	107	51	56	24.43	G25	1.34	20	15	5	34.11
G26	1.43	65	25	40	6.46	G26	1.05	74	24	50	25.97	G26	1.95	83	33	50	20.45
G27	1.84	69	36	33	7.64	G27	0.37	41	6	35	27.47	G27	2.29	78	43	35	26.26
G28	1.07	69	18	51	5.72	G28	2	92	46	46	26.36	G28	3.19	68	56	12	32.57
G29	1.24	47	23	24	9.39	G29	1.09	59	25	34	27.47	G29	2.06	69	40	29	27.32
G3	3.87	61	58	3	13.99	G3	1.12	40	27	13	29.47	G3	2.4	65	48	17	31.3
G30	2.08	92	43	49	5.87	G30	0.35	36	5	31	27.82	G30	1.39	71	16	55	17.97
G31	2.66	92	49	43	6.24	G31	1.94	87	45	42	26.83	G31	1.51	60	21	39	25.85
G32	1.96	70	38	32	7.66	G32	2.63	109	56	53	25.24	G32	3.24	95	57	38	25.94
G33	1.05	47	17	30	8.15	G33	0.64	34	10	24	28.34	G33	0.47	27	1	26	28.85
G34	1.56	88	28	60	3.71	G34	0.78	75	15	60	23.25	G34	2	95	38	57	17.2
G35	1.65	80	33	47	6.01	G35	2.24	78	53	25	28.32	G35	2.36	93	47	46	21.97
G36	1.51	81	26	55	4.68	G36	3	98	59	39	27.16	G36	1.41	70	17	53	20.07
G37	1.57	85	29	56	4.58	G37	0.76	19	14	5	30.9	G37	2.99	108	54	54	19.72
G38	1.24	60	24	36	7.24	G38	1.77	50	43	7	30.15	G38	1.09	40	8	32	26.49
G39	0.48	63	6	57	4.31	G39	0.83	38	16	22	28.43	G39	1.45	61	19	42	24.76
G4	4.84	66	60	6	13.53	G4	0.73	52	12	40	27.13	G4	1.56	38	23	15	31.94
G40	1.68	78	34	44	6.08	G40	0.43	30	7	23	28.36	G40	2.65	94	50	44	23.4
G41	2.6	82	48	34	7.43	G41	1.52	63	36	27	28.27	G41	1.99	55	37	18	31.18
G42	1.64	90	32	58	4.23	G42	1.75	93	41	52	25.29	G42	1.76	88	29	59	15.99
G43	1.55	73	27	46	6.02	G43	2.3	95	54	41	26.87	G43	2.11	82	42	40	25.73
G44	1.21	53	22	31	7.9	G44	2.46	102	55	47	26.3	G44	0.51	58	2	56	17.38
G45	2.07	92	42	50	5.79	G45	0.32	48	4	44	26.58	G45	0.96	29	5	24	29.43
G46	2.16	71	44	27	8.88	G46	1.1	81	26	55	24.47	G46	1.6	46	25	21	30.44
G47	0.75	46	7	39	6.87	G47	0.68	65	11	54	25.04	G47	1.17	48	11	37	26.24
G48	1.87	55	37	18	10.59	G48	1.34	65	33	32	27.55	G48	2.3	69	44	25	28.99
G49	0.46	31	5	26	8.98	G49	2.1	78	48	30	27.94	G49	1.7	35	28	7	33.39
G5	3.86	65	57	8	13.21	G5	2.08	104	47	57	24.16	G5	2.71	81	51	30	27.12
G50	1.97	58	39	19	10.34	G50	1.25	58	32	26	28.3	G50	1.95	80	32	48	21.45
G51	1.77	88	35	53	5.23	G51	0.91	70	19	51	25.42	G51	2.33	94	45	49	21.29
G52	3.29	73	52	21	9.78	G52	1.17	34	31	3	31.46	G52	1.44	19	18	1	39.74
G53	1.04	38	16	22	9.42	G53	1.48	49	35	14	29.4	G53	1.98	77	36	41	25.16
G54	3.47	61	56	5	13.66	G54	1.15	51	30	21	28.51	G54	1.97	95	35	60	11.52
G55	3.45	62	55	7	13.37	G55	2.12	87	49	38	27.28	G55	0.85	38	4	34	26.43
G56	0.94	51	13	38	6.91	G56	2.71	76	57	19	28.6	G56	0.51	30	3	27	27.65
G57	0.81	60	8	52	5.41	G57	2.22	85	52	33	27.48	G57	1.32	66	14	52	20.12
G58	0.9	53	11	42	6.34	G58	1.66	87	38	49	26.03	G58	1.82	89	31	58	16.62
G59	0.96	29	14	15	11.13	G59	1.65	54	37	17	28.95	G59	2.1	64	41	23	29.73
G6	1.6	47	31	16	11.04	G6	1.35	43	34	9	30.09	G6	1.01	50	7	43	23.98
G60	3.38	58	54	4	13.83	G60	0.74	33	13	20	28.53	G60	1.69	74	27	47	21.46
G7	2.51	61	47	14	11.21	G7	0.91	38	20	18	28.87	G7	1.81	34	30	4	34.18
G8	3.37	62	53	9	13.16	G8	1.14	57	29	28	28.05	G8	1.27	44	13	31	26.75
G9	1.15	22	21	1	16.15	G9	0.22	6	2	4	31.21	G9	0.97	20	6	14	32.31

ASV, AMMI stability value; G, Genotype; GSI, genotype selection index; RDMC, root dry matter content; FRY, fresh root yield.

## Discussion

In this study, there were seasonal and environmental effects on the performance and stability of cassava genotypes. During the dry season and in areas with little rainfall, CGM attacks tend to be more severe than during the rainy season. High pubescent found on leaves, longevity of leaves, and amount of leaves found on the apex tip of cassava during the peak of dry season reduce CGM severity. Active plant growth was observed with concurrent reduction of the CGM attack during heavy rainfall. Agreeing with the observation of Yaninek et al., 1989, new plant growth is triggered by rainfall, and mites are washed off the leaves during the rainy seasons.

Estimates of broad sense heritability for the traits were found to be relatively low-to-moderate. This is in agreement with the findings of Ezenwaka et al., 2018. This suggests that the traits had non-additive gene action, and the combination of the genotype and environment effects greatly influenced the expression of the traits. However, traits that show strong dependency on non-additive genetic effects can still be enhanced by reciprocal recurrent selection and marker-assisted breeding (Ceballos et al., 2015; Ezenwaka et al., 2018).

The significant negative correlation observed between CGMS and other traits suggests that genotypes with high pubescent leaves, outstanding leaf retention, very good stay green, high biomass, high RDMC, and high FRY tend to be resistant to CGM attack. The negative pattern of the relationship between CGMS and LP is due to the leaf trichomes that restrict the movement of CGM on the leaves, resulting in a reduction of the pest damage to the leaves. Moreover, LP, specifically on immature leaves and shoot tips, has also been reported to harbor *Typhlodromalus aripo* (a phytoseiid predatory mite and CGM natural enemy) (Onzo et al., 2012). LP is a heritable character that effectively suppresses CGM populations in cassava (Hahn et al., 1989). The negative correlations between CGM and yield traits (biomass, FRY and RDMC) may be explained by the negative impact of the pest on yield.

The significant positive correlation between LP and all other traits means that these traits can be selected simultaneously and enhanced through breeding for resistance to CGM. It has been studied that cassava genotypes that have outstanding leaf retention with very good stay green ability play an important role in promoting the survival of *T. aripo* (Zundel et al., 2009) and other phytoseiid predatory mites (Pratt et al., 2003) both in the rainy and dry seasons.

Cassava yield and economic losses due to CGM attack is a serious threat to rural household incomes and global food security. The results of the multivariate regression analysis revealed the negative significance of CGMS on FRY which caused a loss of 20% of average yield approximately equivalent to a current value of 367 USD (135,000 Naira) per hectare. This proves that CGM damage is a significant factor affecting the quality and quantity of yield cassava root.

The combined AMMI analysis of variance of 60 cassava genotypes revealed that there were significant genotypic variations for all the traits indicating the presence of genetic variation in the population. This genetic variability observed indicates opportunity for selection and prospects for enhancing cassava for the traits. The environmental effect was significant for all the traits which justified the importance for multilocational testing to identify best performers for specifically and generally adapted to the environments. There were significant responses of genotype by environment interaction for all the traits. This implies that the genotypes have different adaptations explaining the need to identify and select location specific genotypes. Alternatively, genotype stability analysis (GSI) can be performed to identify genotypes with better response and improved stability over several years and environments (Mutegi et al., 2009). In this study, genotypes performed better in the ideal environments than under stress environments.

The GSI incorporates both genotype stability and high yield, indicating the best method for identifying ideal genotypes. CGMS was evaluated to identify the most stable and resistant genotypes during the growing period of cassava. GSI ranked G31 (IBA131794), G19 (IBA131762), G52 (TMEB778), G38 (IBA131809), and G11 (IBA131748) as the genotypes that combined resistance with stability. They are the most desirable and can be recommended for wider production or as sources of resistance for breeding program. Genotypes G31 (IBA131794), G52 (TMEB778), G11 (IBA131748), and G19 (IBA131762) were found to combine high stability with high FRY, high level of LP, outstanding LR, enhanced SG, and high RDMC and should be used as parents for hybridization with other cassava genotypes to enhance the level of resistance to CGM and yield traits of cassava. Accordingly, GSI ranked genotypes G52 (TMEB778), G31 (IBA131794), G11 (IBA131748), G19 (IBA131762), and G25 (IBA131776) as the most ideal genotypes for fresh root yield across environments. The check variety G52 (TMEB778) was the highest yielder in all the environments; therefore, a greater number of elite genotypes should be assessed in the future in these environments to obtain superior variety.

## Conclusion

Genotype by environment interaction was significant for most of the traits suggesting the need to evaluate the genotypes in several environments before effective selection is made. The study has identified genotypes, G31 (IBA131794), G19 (IBA131762), G52 (TMEB778), and G11 (IBA131748) as most stable and most resistant to CGM which also combine high FRY and other useful agronomic traits, indicating that these traits can be combined in cassava as preferred by farmers. These genotypes can be tested in more environments to determine their adaptability and potential recommendation for release to farmers for cultivation. Umudike location displayed a low pest pressure followed by Igbariam location which showed a moderately high pest pressure, then Otobi location appeared to be the highest pest pressure zone. However, the research has helped to improve food security in Nigeria and elsewhere where cassava is grown through the identification of CGM resistant genotypes that also have high FRY and RDMC. CGM resistance, high FRY, and RDMC are the lead farmer-preferred traits. The enhancement of these traits through plant breeding is also likely to increase farmers’ adoption of new genotypes.

## Data Availability Statement

The datasets presented in this study can be found in online repositories. The names of the repository/repositories and accession number(s) can be found below: www.cassavabase.org.

## Author Contributions

LJ and CE contributed to conception and design of the study. LJ performed the statistical analysis and wrote the first draft of the manuscript. AD, IA, ED, EB, JO, and CE reviewed, edited and supervised the study. All authors contributed to the article and approved the submitted version.

## Funding

This research was supported by NextGen Cassava Breeding project through Bill and Melinda Gates Foundation and UKAid (Grant 1048542, http://www.gatesfoundation.org) and from the NRCRI, Umudike, Nigeria.

## Conflict of Interest

The authors declare that the research was conducted in the absence of any commercial or financial relationships that could be construed as a potential conflict of interest.
